# Perceived Stress in the Primary Caregivers of Adolescents with Asthma: A Cross-Sectional Study

**DOI:** 10.3390/children9111614

**Published:** 2022-10-25

**Authors:** Selene Valero-Moreno, Inmaculada Montoya-Castilla, Konstanze Schoeps, Silvia Postigo-Zegarra, Marián Pérez-Marín

**Affiliations:** 1 Department Personality, Assessment and Psychological Treatments, Faculty of Psychology and Speech Therapy, Universitat de Valencia, Blasco Ibáñez, 21, 46010 Valencia, Spain; 2 Department Psychology, Faculty of Health Sciences, University European University of Valencia, Passeig de l’Albereda, 7, 46010 Valencia, Spain

**Keywords:** family caregivers, asthma, emotional stress, caregiver burden, adolescence

## Abstract

This study aims to determine the impact of the disease on the perceived stress levels of caregivers of adolescents with asthma. A total of 140 primary caregivers, whose mean age was 45.43 years (SD = 5.03), of whom 85% were mothers, were assessed using the perceived stress questionnaire (PIP), and medical indicators related to asthma were recorded. Mean comparisons, correlations, and qualitative comparative analysis (QCA) models were used. The results indicated moderate levels of perceived stress in caregivers, no kinship differences were found, and age was negatively associated with perceived stress. QCA models suggested that perceived stress could be explained by a higher frequency of visits, poorer adherence, more frequent daily medication doses, and higher severity of asthma. In conclusion, the development of psychological interventions addressing the subjective overload of the family caregiver may benefit them, increasing their well-being, and in turn help to manage the emotional difficulties of adolescents.

## 1. Introduction

When a chronic illness is diagnosed, the entire family is affected. However, in terms of caregiving tasks, it is implicitly or explicitly expected that one family member becomes the patient’s primary caregiver [1]. Generally, this primary family caregiver [2] is often the mother [3,4]. The primary caregiver is the family member in charge of providing priority physical and emotional support to someone else, in a permanent and committed way. That caregiver in turn becomes the subject of secondary health care, as they are exposed to high physical and psychological burdens, to social, economic, and emotional difficulties, and even to important changes in the way their family functions [5]. The primary caregiver is usually identified during the first visit, becoming an essential point of reference for healthcare professionals when designing the intervention plan and making decisions involving the patient.

Asthma usually entails significant complications in its management, being a disease associated with elements of uncertainty, caution, fear, worry, and helplessness. These features may be associated with the presence of emotional or behavioral problems within families, derived from the process of living with this disease on a chronic basis, and the psychological stress present in family caregivers may exacerbate the symptoms of pediatric patients [6,7]. Stress and family conflict can interfere negatively with treatment adherence, by affecting how a family monitors an adolescent’s symptoms and makes treatment decisions [8]. Studies indicate that parents who are less attentive to their children’s medical needs because they are highly stressed allow greater exposure to risky behaviors such as exposure to smoke, viruses, or other agents that can exacerbate asthma attacks [4,9]. The disruption of family routines caused by the stress of asthma may also contribute to inadequate asthma control [10,11,12,13]. A recently conducted review [14] indicated that caregivers of this type of adolescent face a number of psychosocial and economic challenges. The results showed that caregiving overload can affect caregivers’ mental health, reduce their quality of life, interfere with their sleep, and increase family stress. It can be difficult for caregivers to keep up with work demands when they need to frequently accompany their children on medical visits [10].

Studies indicate that higher asthma severity and poorer asthma control [13] exert negative effects on quality of life in some carers, who show higher levels of stress [10,15,16]. Other medical variables [17,18,19] related to overload include crises or exacerbations [20,21,22] that increase the number of emergency department visits and the number of hospitalizations [10]. These increase the psychosocial stress of caregivers not only because of fear about patients’ asthma symptoms, but also because of caregiving stress and interference with their own daily lives. In addition, caregivers of children with poorly controlled asthma report more days off work and more significant financial burden than those with better controlled asthma [23,24,25]. Higher levels of stress are associated with a great number of psychological problems [7,26] including anxiety and depression in the caregiver, leading to complications in the management of asthma. Very high stress in caregivers is associated with activity limitation and increased occurrence of breathlessness in children with asthma. In addition, it has been observed that the patients of these caregivers were twice as likely to have any asthma symptoms on a daily basis [27]. Meanwhile, demographic variables affect perceived stress levels. Low socioeconomic status [22], younger caregivers [28], marital status of single [29], and being female [30] are variables positively associated with elevated stress levels. 

The present study aimed to analyze levels of perceived stress in caregivers of adolescents with asthma, to assess the impact of the disease on their stress levels. The proposed hypotheses were:

**Hypotheses** **1** **(H1).** *Family caregivers will present high levels of stress.*

**Hypotheses** **2** **(H2).** *Family caregivers who are younger mothers will show higher stress scores.*

**Hypotheses** **3** **(H3).** *Caregivers of adolescents with uncontrolled and more severe asthma and allergic comorbidity will show higher stress scores.*

**Hypotheses** **4** **(H4).** *Caregivers of adolescents with asthma with a higher frequency of visits and higher rate of hospitalization will have higher perceived stress.*

**Hypotheses** **5** **(H5).** *Primary caregivers of adolescents with asthma with a longer time since diagnosis and longer treatment time (considering daily doses) will show higher perceived stress scores.*

## 2. Materials and Methods

### 2.1. Design

This study used a single-pass cross-sectional design. 

### 2.2. Sample and Setting

The procedure consisted of identifying all patients with asthma who attended pediatric pulmonology consultations at a hospital. The inclusion criteria for the pediatric patients were that they were between 12 and 16 years old and had been diagnosed with asthma at least six months previously. This time limit was considered the minimum because chronic illnesses are understood to be those that have a duration of more than three months or that require continuous hospitalization for more than one month, and that are so severe that they interfere with daily activities. The inclusion criterion for caregivers was that they were the patient’s primary caregiver, were in charge of the adolescent’s treatment, and usually accompanied them to medical care. The method of contact was that when they accompanied their child to the medical consultation, if the adolescent met the inclusion criteria, the caregiver was informed of the possibility of participating in the study. The procedure was explained to the caregiver, and then the psychologist made sure that the caregiver was the main caregiver. In that case, if they agreed to participate, they signed the informed consent form and the assessment proceeded.

### 2.3. Measurements

Perceived stress: the Pediatric Inventory for Parents (PIP) was used [3,31], which is a scale assesses the stress of parents caring for a chronically ill child. Optimal reliability coefficients were 0.78 for total frequency and 0.81 for total effort [3]. In the present study, the reliability indices were 0.79 and 0.84, respectively.

In addition, an ad hoc questionnaire was used, and the patients’ medical records were reviewed by a pulmonologist to record the data related to medical issues and their interpretation. The variables were:

- Asthma control and severity following the GINA criteria [32]. Asthma control refers to the degree of reduction in the manifestations associated with asthma after treatment has been incorporated. Thus, it contemplates two dimensions: current symptom control and future risk (future interferences in the evolution of the disease). The severity of asthma is conceptualized as the frequency and intensity of symptoms, evaluated by the number of crises, response between crises, nocturnal symptoms, tolerance to physical exercise, the need for rescue medication, and the values obtained through spirometry [8]. The severity assessment can be found in Table 1.

- Medical treatment: the type of treatment taken by asthmatic patients was recorded, differentiating between aerosol, nebulized, and pill therapy.
- Therapeutic adherence: determination of therapeutic adherence was based on medical judgment and recorded medical history.
- Indications: at the time of the evaluation, it was recorded whether treatment was increased, maintained, or reduced.
- Other: diagnosis time (months), frequency of visits, and the number of hospital admissions.

### 2.4. Data Collection Procedure

First, descriptive analyses were performed (mean, standard deviation), then comparison of means was carried out using *t*-tests or one-factor ANOVA, for both of which the effect size was calculated. In the case of the *t*-test, Cohen’s d was used. Values below 0.2 were considered to indicate a small effect size, 0.5 a medium effect size, and 0.8 a high effect size [33]. In the case of one-factor ANOVA, partial eta squared was used. A value of eta squared around 0.01 is usually considered to represent a small effect, around 0.06 indicates a medium effect, and above 0.14 is a large effect [33]. Pearson correlations were also performed between the variables and the age of the caregiver.

Qualitative comparative analysis (QCA) is a technique for exploring the different conditions that may contribute to explaining a given result. Such models are based on Boolean logic, whereby the combination of different variables can lead to the same result. Therefore, in these cases, the ways in which the variables combine (known as the conditions in terms of this methodology) are more important than the individual contributions they make to the results. This methodology allows what is known as equifinality, i.e., arriving at the same result by different combinations or paths. The method is divided into two analyses: necessity analysis, indicating which variables (conditions) must be present for a certain result to be achieved, and sufficiency analysis, referring to which variables can explain the result. Through the QCA models it is possible to obtain what is called coverage, which is similar to the variance explained in the regression models, and also consistency that indicates the model fit [34,35]. In general, one of the advantages of QCA compared with linear models is the possibility of addressing multiple contextual causes in a straightforward manner, with the addition that a specific result can be reached by different routes. Another advantage is the possibility of identifying combinations of multiple causes. Linear models are limited in the number of variables that can be included for analysis, but in QCA models there are multiple possibilities. Furthermore, QCA models allow richer results because they provide more possible combinations compared with regression analysis [36,37]. 

In this study, in order to perform the analysis it was necessary to transform the responses obtained from the adolescents into fuzzy set data. First, as indicated in the literature, all missing values were removed and all constructs or variables were calculated by multiplying their item scores according to the questionnaires. Before running the analysis, the values were recalibrated between 0 and 1. Recalibration is very important because it can affect the result, indicating observations or participants who achieved certain outcomes. When considering only two values, scores were assigned of 0 (without the feature, totally out of the set) and 1 (with the feature, totally within the set), for example in the case of control. However, to recalibrate data for more than two values, the process must consider the three thresholds: the first (0) indicates an observation that this value is totally out of the set (low agreement); the second (0.5) considers it to be at a midpoint, neither in nor out of the set (intermediate level of agreement); and the highest value (1) considers the observation to be totally within the set (high level of agreement). This process that which the original author indicated should be performed to recalibrate the data. [35]. With continuous or factor variables from survey responses (consisting of different items), it is necessary to enter these three values to perform automatic recalibration of values between 0 and 1. Thus, the literature suggests that to achieve recalibration for continuous variables it is necessary to calculate the 10th, 50th, and 90th percentiles [38]: 10% (low agreement or totally out of the set), 50% (intermediate level of agreement, neither in nor out of the set) and 90% (high agreement or totally within the set). When these transformations were carried out, the sufficiency and necessity analyses discussed above were performed. In this model, a sufficient condition is a combination of different variables or conditions that explains a given outcome, always bearing in mind the principle of equifinality, while a necessary condition must always be present for the result to be produced.

In the present analysis, the variables “Total PIP related to stress generated by the frequency of caregiving situations” and “Total PIP related to stress generated by the strain of caregiving situations” for the primary family caregiver were used as dependent variables. The independent variables were asthma control and severity, time since diagnosis, daily medication doses, adherence, medical indications, and frequency of visits. Descriptive analyses of participants were estimated, then calibration values for fsQCA were calculated, followed by fuzzy-set qualitative-comparative analysis (fsQCA). To obtain stress scores, items were first multiplied and then recalibrated, then necessity and sufficiency analyses were carried out using Claude and Christopher’s (2014) fsQCA 2.5 software [39].

### 2.5. Ethical Considerations

The present study was approved by the UV-INV_ETICA-1226194 ethics committee.

## 3. Results

A total of 140 primary family caregivers of adolescents diagnosed with asthma were studied. The most frequent relationship in the study was that of mother to the pediatric patient, with 85% (n = 119), followed by father, with 15% (n = 21). The mean age of the primary caregivers was 45.43 years (SD = 5.03), ranging from a minimum of 27 to a maximum of 63. For more information on caregivers, see Table 2. Among the patients, 86% had controlled asthma for at least the previous six months, while 14% had uncontrolled asthma. In terms of asthma severity, 60.4% had persistent moderate asthma, 32.3% had frequent episodic asthma, 6% had occasional episodic asthma, and 1.3% had severe asthma. 

### 3.1. Levels of Perceived Stress

In general, higher scores were observed in the stress scale values for frequency (number of times they were exposed to stressful caregiving situations) than in the stress scales associated with effort (discomfort or effort involved in dealing with caregiving situations). Moreover, moderate scores were found in relation to stress levels for most of the subscales, and the totals were above average, which seems to indicate that the main caregivers were in stressful situations associated with asthma. The subscales with the highest scores were medical care (referring to situations involving accompanying the patient, dealing with treatment changes, etc.) and communication (situations involving communication about illness-related issues with health staff, family members, and the patients themselves). Meanwhile, despite lower scores, moderate levels of stress were also found for the subscales of emotional distress (relating to uncertainty about the future of the disease or the emotional discomfort the caregiver experienced living with the disease, including lack of time for self-care) and family role (situations regarding changes at work, within the family system, in a couple’s relationship) (Table 3).

### 3.2. Socio-Demographic Variables and Perceived Stress

Observing the sample of caregivers of adolescents with asthma, no differences were found for the kinship variable, although the considerable majority of our sample were mothers. 

On the other hand, the associations between age and the study variables were low to moderate. Statistically significant and negative relationships were found between age and the subscales “communication frequency” (r = −0.25; *p* = 0.02), communication effort” (r = −0.26; *p* = 0.02), “care effort” (r = −0.22; *p* = 0.04), “family role effort” (r = −0.22; *p* = 0.04), “emotional distress effort” (r = −0.24; *p* = 0.03), and also for “total frequency” (r = −0.24; *p* = 0.03), and “total effort” (r = −0.26; *p* = 0.02) according to the stress questionnaire (PIP). Therefore, the younger the age of the caregivers, the higher was their overall perceived stress levels in all sub-dimensions.

### 3.3. Impact of Asthma on Perceived Stress

#### 3.3.1. Comparison of Means and Association of Variables

For asthma control and severity, the type of allergy, its degree of control, and whether the patient was receiving immunotherapy, no statistically significant differences in perceived stress levels were found.

Overall, differences were observed in caregivers of adolescents with asthma that included the presence of allergic symptomatology. These caregivers showed higher levels of stress, especially in the subscales of communication (t = 3.79; *p* = 0.001; d = 0.89), medical care (t = 2.93; *p* = 0.01; d = 0.69), and family role (t = 2.68; *p* = 0.01; d = 0.64). Differences were apparent for therapeutic adherence, particularly in the family role subscale (F = 7.50; *p* = 0.001; η^2^ = 0.14), indicating that caregivers whose patients exhibited poorer adherence experienced higher stress levels. In turn, for caregivers who attended consultations more frequently (every four months), higher stress levels were shown on the communication subscale (F = 6.97; *p* = 0.001; η^2^ = 0.15) compared with those who attended every six months or every year. Differences were found in terms of indications at the time of evaluation; when medical treatment was intensified, the main family caregiver showed higher levels of perceived stress, especially on the subscales of family role (F = 4.29; *p* = 0.02; η^2^ = 0.09), emotional distress (F = 3.07; *p* = 0.05; η^2^ = 0.07), and communication (F = 3.37; *p* = 0.04; η^2^ = 0.08), compared with those whose children had undergone reductions in medical treatment.

Meanwhile, for the medical variable of the number of hospitalizations, positive relationships were observed, but to a low degree. Thus, the higher the number of hospitalizations, the higher the levels of perceived stress in the caregiver, in terms of total stress associated with frequency (r = 0.27; *p* = 0.05) and in the subscales of stress associated with frequency of emotional distress (r = 0.23; *p* = 0.05) and family role (r = 0.24; *p* = 0.05). Finally, the number of daily doses of medication was negatively related to stress levels on the stress-associated communication subscale (r = −0.35; *p* = 0.001), as caregivers whose patients needed to take more medication perceived less stress related to the need to communicate illness-related issues to family members, the patients themselves, or health staff.

#### 3.3.2. QCA Models

In the necessity analysis of the outcome variables, for both high and low levels of perceived stress related to both frequency and effort associated with caregiving tasks, necessary conditions were observed. For high levels of stress associated with frequency, the condition of lower frequency of visits was deemed to be necessary because its consistency was greater than 0.90. In the case of low levels of stress associated with frequency and effort, the only necessary condition was good adherence. In the sufficiency analysis, the models for the primary family caregiver’s “perceived stress” provided the results shown in Table 4.

For predicting high levels of stress (Table 5) associated with frequency of caregiving, four pathways were observed that explained 43% of cases with high levels of stress (total consistency = 0.82; total coverage = 0.43). The most relevant pathways predicting these high stress levels included first the combination of longer diagnosis time, higher frequency of visits, higher asthma severity, and controlled asthma, explaining 31% of the high levels (consistency = 0.88; coverage = 0.37). The second most relevant pathway was the interaction between a higher number of daily doses, worse adherence, and controlled asthma, explaining 14% of the high levels (consistency = 0.97; coverage = 0.14). Finally, the third combination explained 12% of these high levels of stress, represented by the combination of a higher number of daily doses, poorer adherence, and being in the process of tapering medication (consistency = 0.97; coverage =0.12).

For predicting low stress levels associated with the frequency of caregiving tasks, eight pathways were observed that explained 58% of cases with low stress levels (overall consistency = 0.90; overall coverage = 0.58). The main pathway predicting these low stress levels was the combination of good adherence, being in the process of tapering medication, lower asthma severity, and controlled asthma (consistency = 0.91; coverage = 0.42). The second most relevant pathway was the interaction between higher frequency of visits, good adherence, and tapering medication (consistency = 0.94; coverage = 0.35), explaining 35% of the cases. Finally, the third combination explained 35% of the low stress levels, combining shorter diagnosis time, good adherence, tapering medication, and lower severity of asthma (consistency = 0.92; coverage = 0.35).

For predicting high stress levels referring to the effort associated with caregiving tasks, six pathways were observed that explained 23% of cases with high stress levels (total consistency = 0.74; total coverage = 0.23). The main pathways predicting these high stress levels included first the combination of a higher number of daily doses, poor adherence, being in relapse, and higher severity but controlled asthma, explaining 12% of the high levels (consistency = 0.78; coverage = 0.12). The second most relevant pathway was the interaction between lower daily doses, shorter diagnosis time, poor adherence, and higher asthma severity, which in combination explained 8% of the high levels (consistency = 0.94; coverage = 0.08). Finally, the third combination explained 8% of the high stress levels, combining short diagnosis time, poor adherence, and higher severity but controlled asthma (consistency = 0.87; coverage = 0.08).

For the prediction of low stress levels referring to the effort associated with caregiving tasks, seven pathways were observed that explained 49% of the cases with low stress levels (total consistency = 0.91; total coverage = 0.49). The most relevant pathway predicting these low stress levels was the combination of longer diagnosis time, lower frequency of visits, good adherence, and tapering medication (consistency = 0.91; coverage = 0.41), explaining 41% of cases. The second most relevant pathway was the interaction between a lower number of daily doses, longer diagnosis time, good adherence, and tapering medication (consistency = 0.93; coverage = 0.26), explaining 26% of cases. The third combination explained 7% of the low stress levels, involving less frequent visits, less time since diagnosis, and lower severity despite a lack of asthma control (consistency = 0.92; coverage = 0.07).

## 4. Discussion

The present study aimed to analyze the levels of perceived stress in primary caregivers of adolescents with asthma, to assess the impact of the disease on them. Therefore, the first hypothesis suggested that family caregivers will present high levels of stress. The literature indicates that caregivers whose children have a chronic illness tend to experience high levels of stress relating to medical care [10,11,12,13]. When children are younger, parents are fully committed to complying with their medical treatment. However, adolescence is the time when most children forget to take their medication. For these parents, having to deal with this situation can increase their sense of overload, often leading to a lack of focus on their own self-care [16]. Our results are therefore consistent with previous studies. Although the stress levels found in our sample were not very high (a possible explanation because the asthma presented by the adolescents was in the control phase), a large percentage of the primary caregivers showed moderate levels of stress. This stress was mainly reflected in situations related to administration or decisions regarding medical treatment, and accompanying adolescents to medical appointments or during medical tests. However, these issues also relate to communication, meaning that parents found it very stressful to talk about the illness with health staff or with other family members. Therefore, the first hypothesis is accepted. It is important to consider the levels of stress displayed by careers, as they directly influence the appropriate management of the disease [6]. In addition, previous studies have indicated that parents who are highly stressed due to the unexpected nature of asthma can sometimes exacerbate their children’s symptoms. Stress and family conflict can interfere with adherence (misuse of rescue medication, failure to identify symptoms of a flare-up, erratic attendance at emergency departments), affecting the way a family monitors symptoms and makes treatment decisions [9,10].

Regarding the variables related to the caregiver (kinship and age), the second hypothesis suggested that family caregivers who are young mothers will show higher stress scores. No differences were observed between fathers and mothers in the results found, so the hypothesis is rejected, contradicting the data presented in previous studies [41,42,43,44]. One possible explanation is that our sample consisted mostly of mothers, so the representation between mothers and fathers was not sufficiently equal to allow the differences to be tested. Although trends are generally changing in today’s society, it is still usually the woman who plays the role of caregiver, so she tends more often to accompany the adolescent to the hospital or on medical visits, and therefore there was a higher percentage of mothers in our sample [4,45]. The results partially supported the hypothesis regarding age, as we found a negative relationship between perceived stress and age. Previous studies [7,16,28] reported differences between variables among younger caregivers, which were partially supported by the present results. Younger carers face a greater number of developmental milestones associated with their normative developmental stage, for example the stress of adolescence, the entry of both partners into the labor market, and the challenges of parenting. In addition, growing older they have experienced stressful life events more often, which can reduce the feeling of stress because they have developed coping strategies.

The third hypothesis was that caregivers of adolescents with uncontrolled and more severe asthma and allergic comorbidity will show higher stress scores. The results showed that asthma severity and asthma control were not variables that influenced the differences between the groups, as indicated by previous studies [10,13,20]. Increased severity and poor control of asthma have negative effects on the quality of life of many caregivers, increasing their stress levels [15]. In addition, an increased number of asthma attacks or exacerbations (lack of asthma control) also affects perceived overload [20,21,22]. Therefore, this hypothesis is not accepted. Most of the study sample were caregivers for patients with controlled asthma at the same severity level, so no significant differences were likely to be observed. However, this is a relevant issue that needs to be addressed in future research. 

In addition, previous studies [6,20] discussed how asthma is sometimes a complicated disease to manage, which can lead to uncertainty and difficulties in its control. Therefore, an increase in the presence of symptoms leading to emergency department visits or hospitalizations could lead to an increase in perceived stress in the caregiver and harm their mental health [10,26,30,43]. The obtained results were in line with these studies, showing that the number of hospitalizations was negatively associated with levels of perceived stress and the frequency of visits, especially for the communication dimension. In other words, having to communicate continuously with health staff or relatives about their adolescent’s illness caused caregivers to feel overloaded. Therefore, the fourth proposed hypothesis, suggesting that caregivers of adolescents with asthma who experienced a higher frequency of visits and higher number of hospitalizations will have higher perceived stress, is accepted.

Finally, the results indicated that differences were found for medical treatment, with higher numbers of daily doses being associated with higher levels of stress, again in the dimension of communication. The results also showed that an increase in medication at the time of evaluation was associated with higher levels of perceived stress in primary caregivers for most of the dimensions. In terms of adherence, poorer adherence (forgetfulness) by the adolescent was associated with the caregiver being highly stressed, especially in the dimension of family role. This indicates that this lack of adherence to treatment caused interference within the family system due to the overload it generated, and was also positively related to hostile conflicts. The obtained results lead to the acceptance of the fifth hypothesis, in line with previous studies [17,18,19]. It is essential to assess stress, as some studies report that the presence of these symptoms is related to the misuse of medication, inadequate inhaler techniques, medication omission, and reduced confidence in children’s symptom control [9], causing a worsening of patient health.

The results of the QCA models suggest that caregiver stress levels were explained by the combination of controlled but severe asthma and poor adherence to treatment (forgetfulness), and sometimes by the number of daily doses or the time since diagnosis. According to the different combinations, the other variables are associated with these. Therefore, it is necessary to conduct further studies in this regard, addressing the medical variables, to determine the risk groups of patients and primary caregivers who may have inadequate adaptation to the disease, for early detection to avoid negative effects on their physical and psychological health.

The main contributions of this study are its demonstration that the impact of asthma on caregivers’ mental health can be analyzed with a novel methodology, combining all variables to reach a common pathway, which has not been achieved in previous studies. However, this study was not without its limitations, as most of the study sample were in a controlled phase of asthma at the same severity level, so the role of severity on emotional adjustment was not effectively studied. This remains a relevant aspect to be addressed in future research. Another aspect to consider regarding the generalizability of the results is that all participants came from hospitals in the Valencian community, and data from other local communities in Spain were not used, so the sample size could be larger. All these aspects will be taken into account in future research.

## 5. Conclusions

In conclusion, caregivers of adolescents with asthma presented moderate levels of stress even in the controlled phase of the disease. Higher stress scores were presented by (1) younger caregivers, (2) caregivers of adolescents with comorbidity with other diseases (allergies), (3) caregivers of adolescents with a number of medical complications (hospitalizations). The results indicate the importance of variables that could modulate the caregiver’s stress response, such as the adolescent’s adherence to treatment and the number of daily doses.

## 6. Clinical Implications

In conclusion, the development of psychological interventions that address subjective overload may benefit family caregivers, increasing their well-being and in turn helping to manage the emotional difficulties of adolescents with asthma, strengthening their physical and mental health, and ensuring better adaptation to the disease for the whole family system, in addition to the importance of multidisciplinary collaboration and shared decision-making. Physicians caring for adolescents with asthma should explain to their caregivers that their own exposure to stress affects their children. Another relevant approach may be to make changes in the administration of treatments (increasing the amount of medication and reducing the number of times a day that it is administered) to facilitate good management and reduce stress overload. Implementing intervention programs such as telehealth could be useful to avoid travel and thereby reduce the stress of hospital visits compounding that of other roles played by the caregiver. Psychoeducational programs could be offered to adolescents and their families to improve compliance and asthma control, and thus quality of life for themselves and their parents. Finally, it is important to share decision-making between the health professional and the patient to decide together the best way to treat the disease. All these proposals could reduce the emotional impact of the illness on the caregiver, reducing their burden.

## Figures and Tables

**Table 1:** Classification of asthma severity in children, according to GEMA.

	Occasional Episodic	Frequent Episodic	Persistent Moderate	Persistent Severe
Episodes	Few hours or days duration, < one every 10–12 weeks. Maximum 4–5 crises/year.	<one every 5–6 weeks. Maximum 6–8 crises/year.	>One every 4–5 weeks	Frequent
Inter-crisis symptoms	Asymptomatic	Asymptomatic	Mild	Frequent
Wheezing	No presence, with good exercise tolerance.	With intense effort.	With moderate effort.	With minimal effort.
Nocturnal symptoms	_	_	≤2 nights per week	>2 nights per week
Relief medication	_	_	≤3 days per week	>3 days per week
Lung function: (FEV1)	>80%	<80%	>70%–<80%	<70%
Variability	<20%	<20%	>20%–<30%	>30%

Retrieved from GINA; FEV1: forced expiratory volume in 1 s; PEF: peak expiratory flow.

**Table 2:** Socio-demographic characteristics of the participants (n = 140).

Kinship					
Mothers % (n)		Fathers % (n)			
85 (119)		15 (21)			
Marital status					
Married % (n)	Divorced % (n)	Single % (n)	Living as a couple % (n)		
79.90 (112)	11.50 (16)	1.50 (2)	7.10 (10)		
Level of education					
Higher education % (n)	Baccalaureate or vocational training % (n)	Basic studies % (n)	Without studies% (n)		
35.90 (50)	22.10 (31)	35.10 (49)	6.90 (10)		
Current Job					
Employed % (n)		Unemployed % (n)			
62.40 (87)		37.60 (53)			
Employment status					
Civil servant % (n)	Permanent contract % (n)	Self-employed % (n)	Temporary contract % (n)	Unemployed without being paid % (n)	Unemployed with being paid % (n)
12.90 (18)	33.30 (47)	22 (31)	3.20 (4)	22 (31)	6.60 (9)

**Table 3:** Descriptive statistics for Pediatric Inventory of Parents scale values of the primary family caregivers (n = 140).

	M	SD	P25	P50	P75	Range	Min.	Max.
Communication—Frequency	8.83	2.30	7	9	10	3–15	3	15
Communication—Effort	8.54	2.44	7	8	11	3–15	3	11
Medical care—Frequency	10.16	3.01	8	10	12	3–15	3	15
Medical care—Effort	5.78	2.94	3	5	8	3–15	3	14
Emotional Distress—Frequency	8.54	2.44	7	8	11	3–15	3	15
Emotional Distress—Effort	7.52	3.20	5	7	10	3–15	3	15
Family role—Frequency	7.54	2.65	5	8	9	3–15	3	14
Family role—Effort	5.78	2.94	3	5	8	3–15	3	14
Total stress—Frequency	34.98	7.87	30	36	40	12–60	14	55
Total Stress—Effort	24.30	9.11	17	23	30	12–60	12	49

M = mean; SD = standard deviation; P25 = percentile 25; P50 = percentile 50; P75 = percentile 75; Min = minimum; Max = maximum.

**Table 4:** Calibration values for the qualitative comparative analysis model predicting perceived caregiver stress (n = 140).

	Stress Frequency	Stress Effort
M	2,099,544.64	422,696.30
SD	7,950,371.49	3,024,536.24
Min.	4	1
Max.	75,000,000	307,200,000
Calibration values		
P10	1050.30	2.60
P50	140,400	1296
P90	3,841,344	267,710.40

M = mean; SD = standard deviation; p25 = percentile 25; P50 = percentile 50; P75 = percentile 75; Min = minimum; Max = maximum.

**Table 5:** Sufficiency analysis for high and low levels of stress in the perceived primary caregiver: medical variables (n = 140).

Frequency of Consistency Cut-Off 1	High Levels of Stress Frequency Consistency Cut-Off 0.79			Low Levels of Stress Frequency Consistency Cut-Off 0.91			High Levels of Stress in Effort Consistency Cut-Off 0.85			Low Levels of Stress in Effort Consistency Cut-Off 0.89		
	1	2	3	1	2	3	1	2	3	1	2	3
Control	●	●		●			●		●			○
Gravity	●			○		○	●	●	●			○
Indications			○	○	○	●	●			○	○	○
Adherence		○	○	●	●	●	○	○	○	●	●	
Visits	●				●					○		○
Time	●					○		○	○	●	●	
Daily Dose		●	●				●	○			○	
Raw coverage	0.31	0.14	0.12	0.42	0.35	0.35	0.12	0.08	0.08	0.41	0.26	0.07
Unique coverage	0.24	0.01	0.02	0.04	0.01	0.01	0.04	0.01	0.01	0.15	0.01	0.02
Consistency	0.77	0.97	0.97	0.91	0.94	0.92	0.78	0.94	0.87	0.91	0.93	0.92
Total consistency			0.82			0.90			0.74			0.90
Total coverage			0.43			0.58			0.23			0.49

● = presence or high levels; ○ = absence or low levels. All paths are consistent because consistency is above 0.74. Expected vector for high stress levels [40]: 0.0.1.0.1.1.1. Expected vector for low stress levels: 1.1.0.1.0.0.0.

## Data Availability

The datasets generated and/or analyzed during the current study are available from the corresponding author on reasonable request.
